# Effect of glycated hemoglobin index and mean arterial pressure on acute ischemic stroke prognosis after intravenous thrombolysis with recombinant tissue plasminogen activator: Erratum

**DOI:** 10.1097/MD.0000000000015739

**Published:** 2019-05-13

In the article, “Effect of glycated hemoglobin index and mean arterial pressure on acute ischemic stroke prognosis after intravenous thrombolysis with recombinant tissue plasminogen activator”,^[1]^ which appears in Volume 97, Issue 49 of *Medicine*, the time range in the first sentence under 3.1. General information should be “From January 2012 to December 2017, 125 AIS patients received rt-PA intravenous thrombolysis.”

## References

[1] Liu S-Y Cao W-F Wu L-F Effect of glycated hemoglobin index and mean arterial pressure on acute ischemic stroke prognosis after intravenous thrombolysis with recombinant tissue plasminogen activator. *Medicine*. 2018 97:e13216. 30544380 10.1097/MD.0000000000013216 PMC6310570

